# Perceived Social Support as a Statistical Indirect Pathway Linking Adverse Childhood Experiences to Risky Behaviors Among University Students: A Cross-Sectional Study

**DOI:** 10.3390/healthcare14172825

**Published:** 2026-09-03

**Authors:** Beray Aydın, Melike Yalçın Gürsoy

**Affiliations:** 1 Malkara State Hospital, 59300 Tekirdağ, Türkiye; 2 Nursing Department, Çanakkale Onsekiz Mart University, 17100 Çanakkale, Türkiye

**Keywords:** adverse childhood experiences, risky behaviors, perceived social support, indirect-effect analysis, university students, public health nursing

## Abstract

**Background/Objectives:** Adverse childhood experiences (ACEs) are a major public health concern associated with long-term health consequences and a range of risky behaviors. This study examined whether perceived social support statistically accounted for part of the association between ACEs and seven risky behavior dimensions among university students. **Methods:** This cross-sectional online survey included 524 students enrolled at a state university. Data were collected using a descriptive information form, the Adverse Childhood Experiences Scale, the Risky Behaviors Scale, and the Multidimensional Scale of Perceived Social Support. Spearman rank correlations, covariate-adjusted multiple linear regression analyses with HC3 robust standard errors, and indirect-effect analyses using Hayes’ PROCESS macro (Model 4; 5000 bootstrap samples) were conducted. **Results:** Overall, 40.8% of participants reported at least one ACE, with psychological abuse being the most frequently reported experience (20.8%). ACE scores were positively associated with all seven risky behavior dimensions (*p* < 0.001 for six dimensions and *p* = 0.004 for substance use). Significant indirect associations through perceived social support were observed for antisocial behaviors, alcohol use, suicidal tendency, and substance use, but not for smoking, eating habits, or school dropout. **Conclusions:** Although the cross-sectional design precludes causal inference, the findings suggest that perceived social support may play a meaningful role in the associations between ACEs and selected risky behaviors among university students.

## 1. Introduction

Adverse childhood experiences (ACEs) refer to negative experiences to which an individual is exposed during the first 18 years of life, including physical, emotional, and sexual abuse, physical and emotional neglect, and household dysfunction [1]. These experiences may exert lifelong effects on an individual’s physical, psychological, and social development and are reported to be associated with various adverse health outcomes later in life [2,3]. A systematic review of 32 studies involving university student populations reported a prevalence of childhood maltreatment of 64.7% [4]. This suggests that adverse childhood experiences represent an important public health concern.

The university years, coinciding with late adolescence and early adulthood, constitute a critical transition period in which identity development continues, autonomy is acquired, and social roles are reconstructed. During this process, separation from family, increasing academic responsibilities, and the strengthening of peer influence may complicate an individual’s adjustment and increase the likelihood of risky behaviors [5]. Risky behaviors are defined as behaviors that increase the risk of illness, injury, and premature death and may also adversely affect an individual’s academic and social life. Antisocial behaviors, smoking, alcohol and substance use, suicidal tendencies, unhealthy eating habits, and school dropout are among the most common risky behaviors in university students [5,6]. According to Jessor’s Problem Behavior Theory, these behaviors do not develop independently of one another; rather, they form a behavioral pattern influenced by common risk and protective factors [6].

The literature consistently reports that ACEs are associated with risky behaviors [2,7]. Prolonged adversity may contribute to vulnerabilities that are associated with greater engagement in risky behaviors [2]. Within the Problem Behavior Theory framework, such behaviors are understood in relation to interacting risk and protective factors [6]. Within this framework, ACEs represent developmental risks, whereas psychosocial resources may be associated with how these risks are expressed [6,8].

Perceived social support refers to the perceived availability of support from family, friends, a significant other, or the wider social environment [9]. The stress-buffering hypothesis proposes that social support may lessen the burden of stressful experiences [10]. Previous studies have linked childhood adversity with lower perceived support and have shown that social support is associated with better adjustment after adversity [8,11,12,13]. Taken together, these findings support examining perceived social support as a possible statistical pathway linking ACEs to risky behaviors. The present study did not compare mediation and moderation models; perceived social support was examined only as a statistical indirect pathway based on the proposed sequence of associations.

Previous studies have linked ACEs with substance use, self-harm, suicidal behavior, and other risky behaviors [2,7]. However, these outcomes are often examined separately. The present study compares seven risky behavior dimensions within the same sample and examines whether the indirect association through perceived social support differs across them. This outcome-specific comparison constitutes the main contribution of the study.

**H1.**  *Adverse childhood experiences are associated with risky behavior subdimensions.*

**H2.**  *Adverse childhood experiences are negatively associated with perceived social support.*

**H3.**  *Perceived social support is negatively associated with risky behavior subdimensions.*

**H4.**  *Perceived social support shows a significant statistical indirect association between adverse childhood experiences and at least some risky behavior subdimensions.*

## 2. Materials and Methods

### 2.1. Design, Setting, and Time

This cross-sectional study was conducted between January 2025 and May 2026 among students attending a state university (Çanakkale Onsekiz Mart University) using an online survey. The study was reported in accordance with the STROBE (Strengthening the Reporting of Observational Studies in Epidemiology) guidelines for cross-sectional studies.

### 2.2. Population and Sample

The study population consisted of students attending the university (*N* = 40,560). The sample size was calculated using Epi Info 7.2. Using a population size of 40,560, a 95% confidence level, a 5% margin of error, and a conservative expected frequency of 50%, the minimum required sample size was calculated as 381. Convenience sampling, a non-probability sampling method, was used. The online survey link was shared in university student groups, and students were asked to forward the link to their class groups. Because the number of students who received or accessed the survey invitation was unknown, a response rate could not be calculated. Eligible participants were actively enrolled university students aged 18 years or older who voluntarily agreed to participate. A total of 532 questionnaires were submitted. Eight incomplete or invalid questionnaires were excluded before analysis, leaving 524 eligible participants in the analytic sample. No item-level missing data were present among the included participants. The excluded questionnaires were unavailable for assessment of missing-data patterns. A sensitivity power analysis for the largest covariate-adjusted multiple regression model (nine predictors, α = 0.05, power = 0.80, *n* = 524) showed that the sample could detect a minimum effect size of Cohen’s *f*^2^ = 0.030, corresponding to *R*^2^ = 0.029.

### 2.3. Data Collection Tools

Data were collected using a descriptive information form and three scales. The descriptive information form included questions on sex, level of education and school, class year, academic achievement, marital status, family income level, geographic region, place where most of life was spent, parental education level, family structure, parents’ cohabitation status, quality of relationship with parents, smoking and alcohol use, diagnosed psychiatric illness, and history of psychological support. Completion of the entire questionnaire, including the descriptive form and all three scales, took approximately 15 min.

**Adverse Childhood Experiences Scale.** Developed by Felitti et al. [1], the Turkish validity and reliability study of the scale was conducted by Gündüz et al. [14]. It consists of 10 dichotomous items. Five items assess abuse and neglect, including physical, emotional, and sexual abuse and physical and emotional neglect; the remaining five items assess household dysfunction. Although the items cover these two broad content areas, the Turkish validation study supported a single-component structure. Therefore, the scale is scored as a single cumulative index rather than as separate subscales. Each “yes” response corresponds to 1 point, and the total score ranges from 0 to 10, with higher scores indicating greater exposure to ACEs. In this study, the KR-20 coefficient for the 10 dichotomous items was 0.69.

**Risky Behaviors Scale.** Developed by Gençtanırım [5], the validated Turkish version of this 60-item, 5-point Likert-type scale was administered. The subdimensions comprise antisocial behaviors (10 items; score range 10–50), alcohol use (9 items; 9–45), smoking (8 items; 8–40), suicidal tendency (12 items; 12–60), eating habits (8 items; 8–40), school dropout (4 items; 4–20), and substance use (9 items; 9–45). The subdimensions are evaluated independently, and no overall total score is calculated; higher scores indicate higher levels of the corresponding risk. In this study, Cronbach’s alpha coefficients for the subdimensions ranged from 0.81 to 0.96. Although these coefficients indicate high internal consistency, possible item redundancy, particularly in subdimensions with the highest coefficients, cannot be excluded. Future studies should consider confirmatory factor analysis to further evaluate the factor structure of the instrument in university student populations.

**Multidimensional Scale of Perceived Social Support.** Developed by Zimet et al. [9], the Turkish validity, reliability, and adaptation study of the scale was conducted by Eker and Arkar [15]. This 7-point Likert-type scale consists of 12 items and has a three-dimensional structure comprising perceived support from family, friends, and a significant other. Each subdimension consists of four items. The total score ranges from 12 to 84, with higher scores indicating greater perceived social support. In this study, Cronbach’s alpha was 0.96 for the total scale, 0.88 for family support, 0.86 for friend support, and 0.88 for significant-other support.

### 2.4. Data Analysis

Data were analyzed using IBM SPSS Statistics version 25.0 (IBM Corp., Armonk, NY, USA). Descriptive statistics included frequency, percentage, mean, and standard deviation. The distributions of the scale and subdimension scores were evaluated using skewness and kurtosis values. Because the ACE score and several risky behavior subdimension scores showed marked non-normality, associations among the scale and subdimension scores were examined using Spearman rank correlation analysis. To control the false discovery rate across the 55 pairwise correlations, the Benjamini–Hochberg procedure was applied. Reliability of the 10 dichotomous ACE items was additionally assessed using the Kuder–Richardson Formula 20 (KR-20).

Multiple linear regression was used because the risky behavior subdimension scores were analyzed as continuous outcomes. Separate multiple linear regression analyses were conducted for each risky behavior subdimension. Each subdimension was entered separately as the dependent variable, while ACEs and total perceived social support were the main independent variables. All models were adjusted for age, sex, family income, psychiatric diagnosis, and history of psychological support. These covariates were selected because of their potential associations with risky behaviors and perceived social support and their possible confounding influence on the estimated associations. Linearity, residual independence, homoscedasticity, multicollinearity, and influential observations were examined. Because heteroscedasticity was detected in several models, HC3 robust standard errors were used. Multicollinearity was assessed using tolerance and variance inflation factor values.

The total perceived social support score was used in the indirect-effect analyses because the primary aim was to examine overall perceived support as a common psychosocial resource across the risky behavior dimensions. The family, friend, and significant-other support subdimensions were examined separately in the descriptive and correlation analyses. Indirect associations were examined using Hayes’ PROCESS Model 4 with 5000 bootstrap samples and the same covariates. An indirect effect was considered statistically significant when its 95% bootstrap confidence interval did not include zero. Fully standardized indirect effects and their 95% bootstrap confidence intervals were also calculated. Statistical significance was set at *p* < 0.05.

### 2.5. Ethical Considerations

The study was conducted in accordance with the principles of the Declaration of Helsinki. Ethical approval (protocol code: 2024-YÖNP-5357; decision no: 17/67; date: 5 December 2024) was obtained from the Ethics Committee of the Institute of Graduate Studies of Çanakkale Onsekiz Mart University, and institutional permission was obtained from the relevant rectorate. Participant consent was obtained via the informed consent form on the first page of the online survey. The necessary permissions for the use of the scales were obtained from the corresponding authors.

## 3. Results

### 3.1. Descriptive Characteristics of Participants

The mean age of the participants was 21.16 ± 2.27; the majority were female (67.0%) and single (74.2%). Most students were enrolled at the undergraduate level (73.7%), with the greatest participation from fourth-year students (36.1%). Most participants rated their academic achievement as moderate (59.7%) and reported that their family income was approximately equal to their expenses (60.1%). The majority did not smoke (65.5%) or use alcohol (74.4%), and most had no diagnosed psychiatric illness (93.9%) or history of psychological support (94.1%) (Table 1).

### 3.2. Descriptive Findings for the Scales

Of the participants, 40.8% (*n* = 214) reported at least one adverse childhood experience. The three most common types were psychological abuse (20.8%), physical abuse (16.8%), and psychological neglect (13.0%) (Table 2).

Mean scores for the subdimensions of the Risky Behaviors Scale are presented in Table 3. Because the number of items and score ranges of the subdimensions differ, the means should be interpreted with reference to each subdimension’s own range. Skewness was greatest for substance use, alcohol use, and antisocial behaviors; the corresponding skewness and kurtosis values are presented in Table 3.

The mean total score of the Multidimensional Scale of Perceived Social Support was 62.92 ± 22.55. Among the subdimensions, the highest mean was for friend support (21.94 ± 7.88) and the lowest for significant-other support (19.17 ± 9.69) (Table 4). Skewness and kurtosis values for the social support dimensions are also shown in Table 4.

### 3.3. Correlations Among Scale Scores

The ACE total score was positively correlated with all risky behavior subdimensions, with correlations ranging from r = 0.199 for substance use to *r* = 0.388 for suicidal tendency (all *p* < 0.01). The ACE total score was positively and significantly correlated with all subdimensions of risky behaviors (antisocial behaviors *r* = 0.354; suicidal tendency *r* = 0.388; *p* < 0.01). While the family support subdimension was negatively and significantly correlated with all risky behavior subdimensions, significant-other support was significantly correlated only with suicidal tendency (*r* = −0.222; *p* < 0.01) (Table 5). After Benjamini–Hochberg correction for 55 pairwise comparisons, all correlations that were significant in the original analysis remained statistically significant.

### 3.4. Multiple Linear Regression Analysis

All covariate-adjusted regression models were statistically significant (*p* < 0.001). VIF values ranged from 1.01 to 1.67 and tolerance values from 0.60 to 0.99, indicating no problematic multicollinearity. Durbin–Watson values ranged from 1.85 to 2.07, and no observation had a Cook’s distance approaching 1. After adjustment, ACEs remained positively associated with all risky behavior subdimensions (β = 0.233–0.348; *p* < 0.001 for six subdimensions and *p* = 0.004 for substance use). Perceived social support was negatively associated with antisocial behaviors, alcohol use, suicidal tendency, and substance use, but not with smoking, eating habits, or school dropout. The adjusted models explained 6.8% to 28.1% of the variance (Table 6).

### 3.5. Indirect-Effect Analysis

In the covariate-adjusted indirect-effect analyses, the 95% bootstrap confidence intervals did not include zero for antisocial behaviors (*B* = 0.168; 95% *CI* = 0.037–0.330), alcohol use (*B* = 0.152; 95% *CI* = 0.014–0.312), suicidal tendency (*B* = 0.362; 95% *CI* = 0.145–0.639), and substance use (*B* = 0.193; 95% *CI* = 0.066–0.354). The indirect associations for smoking, eating habits, and school dropout were not statistically significant because their confidence intervals included zero (Table 7). The fully standardized indirect effects were 0.033 for antisocial behaviors, 0.029 for alcohol use, 0.045 for suicidal tendency, and 0.048 for substance use; their 95% bootstrap confidence intervals excluded zero. Fully standardized indirect effects were not significant for smoking, eating habits, or school dropout.

## 4. Discussion

This study examined the associations among ACEs, perceived social support, and seven risky behavior dimensions. H1, H2, and H3 were supported. H4 was partially supported, as significant indirect associations were observed for antisocial behaviors, alcohol use, suicidal tendency, and substance use, but not for smoking, eating habits, or school dropout. These results describe concurrent statistical associations and do not establish temporal or causal relationships.

Among university students, ACEs have been associated with suicidal ideation and risky behaviors [16], within a broader literature demonstrating the widespread prevalence of childhood maltreatment across populations [17]. Globally, suicide was the third leading cause of death among individuals aged 15–29 years in 2021 [18], and suicidal ideation has also been emphasized as an important mental health concern among university students [19]. The smoking rate observed in this study was generally similar to rates reported among university students in Türkiye [20] and in international samples [21].

Participants’ perceived social support levels were generally high, with the highest score for friend support and the lowest for significant-other support. This pattern may reflect the importance of peer support during university life, as support from friends has been associated with depressive symptoms and quality of life among university students [22]. The high family support scores also suggest that students maintain ties with their families during the transition to university. The findings are consistent with recent systematic reviews reporting that higher perceived social support is associated with better mental health and psychological adjustment [23].

In this study, ACEs were positively associated with all subdimensions of risky behaviors. This finding is consistent with evidence linking ACEs to difficulties in coping and self-regulation, which may contribute to greater engagement in risky behaviors [2]. Felitti et al. showed that the risk of smoking, alcohol and substance use, and suicide attempts increased in individuals with a history of ACEs [1], while Hughes et al. showed that the risk of problematic substance use and self-directed violence reached its highest level in individuals exposed to four or more ACEs [2]. Studies conducted with university students in Türkiye have also reported associations between childhood adversity and risky alcohol or substance use [24,25]. This pattern is consistent with Problem Behavior Theory, which views different risky behaviors as related outcomes shaped by shared risk and protective factors [6].

Perceived social support was generally inversely associated with risky behaviors, and family support showed the most consistent correlations across the seven subdimensions. This finding is consistent with studies linking social support with lower risky behavior and better adjustment [26,27,28] and with the stress-buffering hypothesis [10]. However, the correlations and modest effect sizes do not show that social support prevents risky behavior.

Perceived social support showed significant indirect associations between ACEs and antisocial behaviors, alcohol use, suicidal tendency, and substance use. Similar indirect or protective associations have been reported between childhood adversity, social support, and later mental health outcomes [8,11,29]. The observed pattern remained after adjustment for age, sex, family income, psychiatric diagnosis, and history of psychological support. However, the modest explained variance and indirect effect sizes indicate that perceived social support accounts for only part of the associations between ACEs and risky behaviors. No significant indirect association was found for smoking, eating habits, or school dropout. The absence of significant indirect associations for smoking and eating habits may reflect the influence of behavior-specific factors, such as dependence, habitual patterns, body-image concerns, and sociocultural influences, that are not fully captured by overall perceived social support. These possible explanations should be examined in future studies. Thus, the role of perceived social support was not uniform across risky behavior dimensions. The fully standardized indirect effects were small in magnitude (0.029–0.048), suggesting limited practical importance despite their statistical significance. These findings extend previous work by showing that the indirect association through perceived social support differs across risky behavior dimensions. This outcome-specific pattern is consistent with Problem Behavior Theory, which recognizes both shared risks and behavior-specific influences [6].

### Limitations

This study has several limitations. First, the cross-sectional design does not permit establishing temporal precedence or causality among the variables; therefore, the indirect-effect findings should be interpreted as concurrent statistical associations rather than evidence of a causal mechanism. Mediation analyses conducted with cross-sectional data may yield biased estimates of longitudinal parameters [30], and the findings should be verified with longitudinal designs. Second, all variables were assessed simultaneously using self-report measures, increasing the potential for common method bias, recall bias, and social desirability bias [31]. Third, although the models were adjusted for age, sex, family income, psychiatric diagnosis, and history of psychological support, residual confounding by unmeasured factors cannot be excluded. The Benjamini–Hochberg correction reduced the risk of false-positive correlation findings, but the multiple outcome-specific models should still be interpreted cautiously. Fourth, perceived social support was modeled using the total score; the stronger correlations for family support indicate that subdimension-specific pathways warrant further examination. Fifth, the use of a single-university convenience sample limits generalizability and may have introduced selection bias, as students who were more active in university or class groups may have been more likely to participate. Sixth, although psychological counseling services were available at the university, specific referral or support information was not provided within the online survey. This should be considered when designing future studies involving similarly sensitive topics. Finally, the KR-20 coefficient of 0.69 for the dichotomous ACE index was slightly below the commonly accepted threshold of 0.70. This suboptimal reliability may have attenuated the observed strength of associations involving ACEs; therefore, these estimates should be interpreted cautiously. In addition, although the sensitivity power analysis indicated that the study could detect effects of *f*^2^ = 0.030 or greater, the study may have been underpowered to detect smaller true effects, potentially increasing the risk of Type II error.

## 5. Conclusions

After adjustment for relevant covariates, ACEs remained associated with all seven risky behavior dimensions, while significant indirect associations through perceived social support were observed only for antisocial behaviors, alcohol use, suicidal tendency, and substance use. This outcome-specific pattern suggests that risky behaviors should not be treated as a single, uniform outcome. University health services may consider assessing students’ available social support and, where appropriate, facilitating referrals to counseling and peer-support resources. These implications should be interpreted cautiously because no intervention was tested in the present study. Because the study was cross-sectional and the effects were modest, the findings should not be interpreted as evidence that increasing social support will directly reduce risky behavior. Longitudinal and multicenter studies are needed to examine temporal relationships and the separate contributions of family, friend, and significant-other support.

## Figures and Tables

**Table 1:** Descriptive characteristics of the participants (*n* = 524).

Variable	Group	*n*	%
Sex	Female	351	67.0
	Male	170	32.4
	Prefer not to say	3	0.6
Level of education	Associate	138	26.3
	Undergraduate	386	73.7
Class year	1st year	172	32.8
	2nd year	109	20.8
	3rd year	54	10.3
	4th year	189	36.1
Academic achievement	Poor	20	3.8
	Moderate	313	59.7
	Good	191	36.5
Marital status	Single	389	74.2
	Married	14	2.7
	In a relationship	121	23.1
Family income level	Income < expenses	104	19.9
	Income = expenses	315	60.1
	Income > expenses	105	20.0
Relationship with parents	Poor	14	2.7
	Moderate	173	33.0
	Good	337	64.3
Smoking	Yes	181	34.5
	No	343	65.5
Alcohol use	Yes	134	25.6
	No	390	74.4
Psychiatric diagnosis	Yes	32	6.1
	No	492	93.9
History of psychological support	Yes	31	5.9
	No	493	94.1

**Table 2:** Distribution of Adverse Childhood Experiences Scale scores (*n* = 524).

Adverse Childhood Experience	*n*	%
Child abuse/neglect		
Psychological abuse	109	20.8
Physical abuse	88	16.8
Sexual abuse	36	6.9
Psychological neglect	68	13.0
Physical neglect	12	2.3
Household dysfunction		
Parental divorce/death	61	11.6
Witnessing domestic violence	30	5.7
Household member with substance dependence	36	6.9
Household member with mental illness	34	6.5
Incarceration of a family member	23	4.4

**Table 3:** Distribution of Risky Behaviors Scale subdimension scores (*n* = 524).

Subdimension	Min.	Max.	Mean	SD	Skewness	Kurtosis
Antisocial behaviors	10	50	18.15	7.49	1.710	3.424
Alcohol use	9	45	12.95	7.69	2.285	4.670
Smoking	8	40	17.40	9.58	0.831	−0.671
Suicidal tendency	12	60	23.70	11.90	1.079	0.435
Eating habits	8	40	19.29	8.09	0.487	−0.384
School dropout	4	20	7.60	4.03	1.059	0.140
Substance use	9	45	11.09	6.00	3.679	13.765

SD = standard deviation.

**Table 4:** Distribution of Multidimensional Scale of Perceived Social Support scores (*n* = 524).

Dimension	Min.	Max.	Mean	SD	Skewness	Kurtosis
Family support	4	28	21.79	8.06	−1.064	−0.305
Friend support	4	28	21.94	7.88	−1.118	−0.145
Significant-other support	4	28	19.17	9.69	−0.538	−1.396
Total score	12	84	62.92	22.55	−0.912	−0.355

SD = standard deviation.

**Table 5:** Spearman correlation analysis among scale scores (*n* = 524).

	1	2	3	4	5	6	7	8	9	10	11
1. Adverse childhood experiences	1										
2. Family support	−0.279 **	1									
3. Friend support	−0.164 **	0.757 **	1								
4. Significant-other support	−0.133 **	0.612 **	0.560 **	1							
5. Antisocial behaviors	0.354 **	−0.291 **	−0.184 **	−0.073	1						
6. Alcohol use	0.222 **	−0.167 **	−0.094 *	−0.006	0.465 **	1					
7. Smoking	0.228 **	−0.130 **	−0.075	−0.010	0.483 **	0.445 **	1				
8. Suicidal tendency	0.388 **	−0.305 **	−0.243 **	−0.222 **	0.522 **	0.304 **	0.366 **	1			
9. Eating habits	0.223 **	−0.126 **	−0.063	−0.051	0.494 **	0.225 **	0.472 **	0.543 **	1		
10. School dropout	0.207 **	−0.193 **	−0.163 **	−0.046	0.448 **	0.292 **	0.421 **	0.525 **	0.488 **	1	
11. Substance use	0.199 **	−0.178 **	−0.169 **	−0.078	0.355 **	0.429 **	0.309 **	0.288 **	0.250 **	0.402 **	1

* *p* < 0.05; ** *p* < 0.01. Significance was retained after Benjamini–Hochberg false-discovery-rate correction.

**Table 6:** Covariate-adjusted associations of ACEs and perceived social support with risky behaviors.

Dependent Variable	*R* ^2^	Adj. *R*^2^	*F*	ACE β	*p*	PSS β	*p*
Antisocial behaviors	0.167	0.152	11.41	0.308	<0.001	−0.140	0.014
Alcohol use	0.132	0.117	8.69	0.251	<0.001	−0.123	0.031
Smoking	0.126	0.111	8.23	0.238	<0.001	0.003	0.949
Suicidal tendency	0.281	0.269	22.37	0.348	<0.001	−0.190	<0.001
Eating habits	0.068	0.051	4.15	0.233	<0.001	−0.022	0.672
School dropout	0.119	0.103	7.70	0.250	<0.001	−0.040	0.418
Substance use	0.124	0.109	8.08	0.238	0.004	−0.200	0.002

ACEs = adverse childhood experiences; PSS = perceived social support; β = standardized beta coefficient; Adj. *R*^2^ = adjusted R squared. Models were adjusted for age, sex, family income, psychiatric diagnosis, and history of psychological support. HC3 robust standard errors were used.

**Table 7:** Covariate-adjusted indirect associations through perceived social support.

Dependent Variable	Total Effect (*B*)	Direct Effect (*B*)	Indirect Effect (*B*)	95% *CI*	Fully Standardized Indirect Effect	95% *CI*
Antisocial behaviors	1.724	1.556	0.168	0.037–0.330	0.033	0.008–0.063
Alcohol use	1.450	1.299	0.152	0.014–0.312	0.029	0.003–0.058
Smoking	1.532	1.536	−0.005	−0.152–0.135	−0.001	−0.023–0.021
Suicidal tendency	3.149	2.787	0.362	0.145–0.639	0.045	0.018–0.079
Eating habits	1.298	1.270	0.028	−0.102–0.170	0.005	−0.019–0.031
School dropout	0.706	0.680	0.026	−0.038–0.094	0.010	−0.014–0.034
Substance use	1.153	0.961	0.193	0.066–0.354	0.048	0.018–0.082

*CI* = confidence interval; *B* = unstandardized coefficient. Models were adjusted for age, sex, family income, psychiatric diagnosis, and history of psychological support. A total of 5000 bootstrap samples were used. The fully standardized indirect effect was calculated as the unstandardized indirect effect multiplied by the standard deviation of ACEs and divided by the standard deviation of the outcome.

## Data Availability

The data presented in this study are available upon request from the corresponding author due to privacy restrictions.
